# 291. Epidemiology of Candidemia Rates during COVID-19 and Comparison of Outcomes in Candidemia Between COVID-19 and Non-COVID-19 Patients

**DOI:** 10.1093/ofid/ofab466.493

**Published:** 2021-12-04

**Authors:** Angela Beatriz Cruz, Jennifer LeRose, Kenisha J Evans, Monica Meyer, Teena Chopra, Teena Chopra

**Affiliations:** 1 Detroit Medical Center - Wayne State University, Detroit, Michigan; 2 Michigan State University College of Osteopathic Medicine, Beverly Hills, Michigan; 3 DETROIT MEDICAL CENTER, DETROIT, Michigan; 4 Wayne State University School of Medicine, Detroit, Michigan; 5 Detroit Medical Center, Wayne State University, Detroit, MI

## Abstract

**Background:**

Fungemia is associated with high rates of morbidity, mortality and increase in length of hospital stay. Several studies have recognized increased rates of candidemia since the COVID-19 pandemic.

**Methods:**

A retrospective cohort study was conducted at a tertiary healthcare system in Detroit, Michigan to evaluate the impact of the COVID-19 pandemic on incidence of candidemia. The “pre COVID-19” timeframe was defined as January – May 2019 while the “during COVID-19” timeframe was January – May 2020. To compare incidence and patient characteristics between cohorts, t-tests and chi-square analysis was used. Additional sub-analysis was performed in candidemia patients during COVID-19 timeframe comparing outcomes of patients based on COVID-19 status. A Fisher Exact and Satterthwaite Test were used for analysis of categorical and continuous variables, respectively.

**Results:**

Overall, 46 cases of candidemia were identified in both the pre COVID-19 and during COVID-19 periods. Pre COVID-19, the average number of cases was 3.0 ± 1.2 per month. The incidence more than doubled during COVID-19 to 6.2 ± 4.2 cases per month (*p* = 0.14) (Figure 1). No significant differences in patient demographics were detected between cohorts, however, patients in the COVID-19 cohort had higher rates of corticosteroid use, mechanical ventilation and vasopressors (Table 1). In the 2020 period, 31 patients developed candidemia and 12 (38.7%) patients tested SARS-CoV-2 positive. On average, COVID-19 patients developed candidemia 12.1 days from admission, compared to 17.8 days in the COVID-19 negative cohort (*p* = 0.340). Additionally, COVID-19 patients with candidemia coinfection were significantly more likely to expire; 83.3% (n=10) COVID-19 patients expired compared to 36.8 (n=7) in the COVID-19 negative cohort (*p* = 0.025) (Table 2).

Figure 1. Incidence of Candidemia in the Pre-COVID-19 (January 2019 – May 2019) and During COVID-19 (January 2020-May 2020) periods

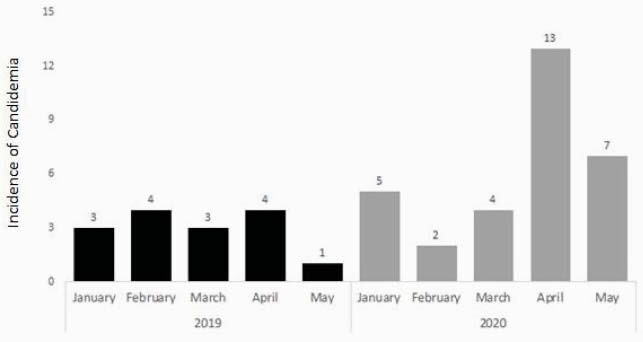

Table 1. Characteristics of Candidemia patients in the pre-COVID (January 2019-May 2019) and during-COVID periods (January 2020-May 2020)

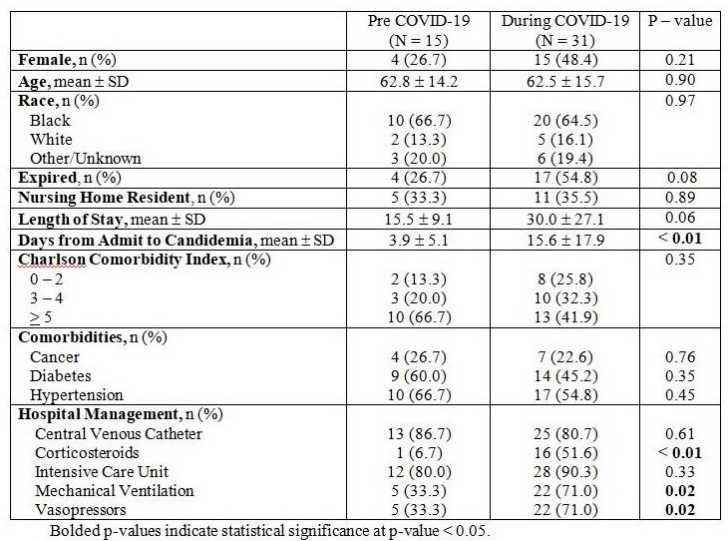

Table 2. Characteristics of Candidemia patients in the SARS-COV-2 negative and SARS-COV-2 positive cohorts from January 2020-May 2020

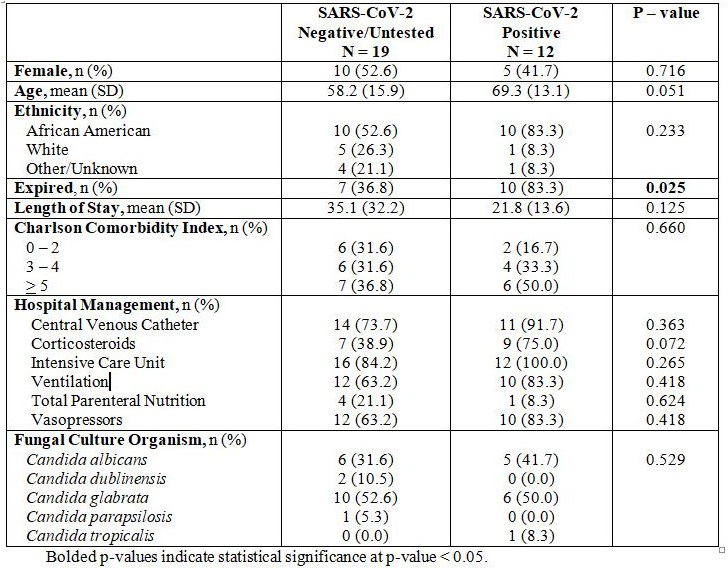

**Conclusion:**

The prevalence of fungemia markedly increased during the COVID-19 surge. Increased use of corticosteroids and broad spectrum antimicrobials, prolonged use of central venous catheters and prolonged ICU length of stay likely contributed to this increase. Patients who developed candidemia co-infection with COVID-19 were found to have poorer outcomes as compared to those who were SARS-CoV-2 negative or untested.

**Disclosures:**

**All Authors**: No reported disclosures

